# Chicago Hospital for Women and Children

**Published:** 1866-10

**Authors:** 


### CHICAGO HOSPITAL FOR WOMEN AND CHILDREN.

We have received the Annual Report of this most excellent charity.

The Hospital for Women and Children is an Institution founded and supported by benevolent individuals of Chicago and vicinity for the following objects:

1st, To afford a home for women and children among the respectable poor in need of medical and surgical treatment;

2d, To sustain a free dispensary for the benefit of the same class;

3d, As incidental to the above, to train competent nurses from this class of our population.

The Institution is under the direction of a Board of Trustees, composed of eleven gentlemen and fifteen ladies, all well known for their kind efforts to aid the needy and especially to relieve their sufferings.

The patients are under the immediate care of Miss M. H. Thompson, M. D., formerly assistant to the New York Infirmary for Women and Children, who is not only Resident Physician but also serves as Matron and Superintendent.

The following is the Board of Consulting Physicians and Surgeons:

| *Physicians.* | *Surgeons.* | *Accoucheurs.* |
|---|---|---|
| W. G. Dyas, M.D. | A. Fisher, M.D. | Thos. Bevan, M.D. |
| C. G. Smith, M.D. | E. Powell, M.D. | E. Marguerat, M.D. |
| S. C. Blake, M.D. | T. D. Fitch, M.D. | H. W. Jones, M.D. |

The Resident Physician has received the warmest approbation of the Trustees, and has the full confidence of the Board of Consulting Physicians.

During the past year 203 patients have been received into the Hospital, and 544 have been treated at the Dispensary. There were 36 obstetrical cases in the Hospital.

This Institution is similar in its objects and organization to the Infirmary for Indigent Women and Children in New York city. It is well known that this charity, under the immediate direction of Drs. Elizabeth and Emily Blackwell, is in a flourishing condition, having for its friends some of the most distinguished physicians of New York. In 1862, Drs. Valentine Mott, Willard Parker, G. A. Sabine, George Camman and John Watson were Consulting Physicians.

---

A limited number of students will be admitted to St. Luke's Hospital, once a week, for clinical instruction.

